# The effect of prophylactic FVIII infusion combined with personalized rehabilitation on joint health and quality of life in children with hemophilia

**DOI:** 10.3389/fped.2025.1578617

**Published:** 2025-07-10

**Authors:** Yaojin Sun, Qian Zhang, Xiaojie Gu, Yongjun Fang, Jie Huang, Heng Zhang

**Affiliations:** ^1^ Department of Rehabilitation Medicine, Children’s Hospital of Nanjing Medical University, Nanjing, China; ^2^ Department of Ultrasound, Children’s Hospital of Nanjing Medical University, Nanjing, China; ^3^ Department of Hematology, Children’s Hospital of Nanjing Medical University, Nanjing, China

**Keywords:** hemophilia, joint health, pediatric patients, MDT, rehabilitation therapy

## Abstract

**Background:**

Hemophilia, a genetic disorder characterized by deficiencies in clotting factors VIII or IX, often results in joint and muscle bleeding, leading to hemophilic arthropathy and reduced quality of life. While multidisciplinary teams (MDTs) are widely used in hemophilia management, personalized rehabilitation strategies for pediatric patients remain underexplored. This study aimed to evaluate a 12-week individualized rehabilitation program targeting joint health in pediatric hemophilia patients.

**Methods:**

This prospective study enrolled 59 children with hemophilia, implementing tailored rehabilitation plans based on specific joint pathologies. Assessments before and after treatment included joint ultrasound examinations (HEAD-US-C), Hemophilia Joint Health Scores (HJHS 2.1), and the CHO-KLAT tool for quality of life. Bleeding frequency was also recorded.

**Results:**

Post-treatment, median HEAD-US-C scores decreased from 5.00 to 1.00 (*P* < 0.001), and HJHS 2.1 scores decreased from 9.00 to 2.00 (*P* < 0.001). CHO-KLAT scores increased from 71.51 to 79.15 (*P* < 0.001). Median bleeding episodes dropped from 3.00 to 0.00 (*P* < 0.001), indicating enhanced joint health and reduced bleeding frequency.

**Conclusion:**

Regular prophylactic FVIII administration combined with individualized rehabilitation significantly reduces joint bleeding and enhances joint function and quality of life in children with hemophilia. The MDT approach is integral to comprehensive care, but further studies are needed to assess the long-term efficacy and safety of this therapeutic strategy.

## 1 Introduction

Hemophilia is a classic X-linked recessive hereditary disorder, with marked gender disparities and age-related clustering in its epidemiological features. Globally, the incidence among male newborns is approximately 1 in 5,000 to 1 in 10,000. Of these, severe cases (FVIII activity <1%) account for 25%–30%, moderate (1%–5%) for 15%–20%, and mild (5%–40%) for 50%–60% (1). According to the World Federation of Hemophilia (WFH) 2023 report, over 190,000 patients are registered worldwide, with children under 18 comprising 45%—making them the population bearing the highest disease burden.

Childhood represents a critical window for early intervention in hemophilic joint disease. Frequent intra-articular bleeding can lead to hemophilic arthropathy, a complication that severely compromises quality of life (2–4). Joint complications—including chronic synovitis, hemosiderin deposition, cartilage degeneration, and eventual joint destruction—further exacerbate functional impairment in affected individuals (5).

Conventional rehabilitation models face several limitations. Widely used clinical guidelines such as the Physiotherapy Guidelines for Hemophilia are primarily based on adult data and lack appropriate adjustments for the unique growth and developmental needs of children. Moreover, the safety of rehabilitation heavily depends on adequate clotting factor levels. At the household level, adherence is challenged by limited professional guidance and the high time demands placed on caregivers. There is an urgent need for innovative intervention systems tailored to pediatric patients (6).

Currently, a multidisciplinary team (MDT) approach, comprising hemophilia specialists collaborating closely with the rehabilitation, radiology, orthopedics, laboratory, and pharmacy departments, is widely advocated in hemophilia treatment centers worldwide (7, 8). While rehabilitation therapy for adult hemophilia patients has made considerable strides, the application of individualized rehabilitation therapy in pediatric patients remains underreported (9).

This study aimed to analyze the MDT collaborative model in pediatric hemophilia treatment and to evaluate its impact on the joint structure, function, and quality of life in children with hemophilia, integrating individualized rehabilitation therapy tailored to address hemophilic arthropathy. By combining personalized rehabilitation strategies with specific interventions targeting joint pathology, such as range-of-motion exercises, strengthening exercises, and joint-protection techniques, we sought to mitigate the progression of hemophilic arthropathy and to improve overall outcomes for pediatric hemophilia patients. Through observation of changes before and after treatment, we aimed to determine the therapeutic effects of this approach, with the goal of providing clinical treatment strategies to enhance joint health and quality of life for children with hemophilia.

Currently, there are numerous studies focusing on rehabilitation to improve joint health in hemophilia patients. For example, a study evaluated the effectiveness of a combined protocol of therapeutic exercise and cognitive-behavioral therapy on the functionality, pain, and joint health of people with hemophilia who experience arthropathy and chronic pain (10). This study found that combining conventional physical therapy with cognitive-behavioral therapy led to partial improvements in joint function, pain perception, and overall joint health. In addition, the participants expressed satisfaction with the treatment. Another prospective study performed in Portugal applied various psychological therapies to adult hemophilia patients, demonstrating effectiveness in pain management, emotional regulation, and quality-of-life enhancement (11).

Our research combined conventional physical therapy, home training, and online rehabilitation support to manage joint health in children with hemophilia comprehensively, while also addressing the psychological needs of both children and their parents. Using appropriate clotting factor levels and joint ultrasound as support, we applied an individualized intervention model for children with hemophilia, anchored by the joint health evaluation results of the Hemophilia Joint Health Scores (HJHS 2.1).

## 2 Methods

### 2.1 Inclusion and exclusion criteria

The criteria for inclusion in this study were as follows: The participants must meet the diagnostic criteria for hemophilia outlined in the “Chinese Expert Consensus on Diagnosis and Treatment of Hemophilia (2017 Edition),” with negative inhibitor status (12). They must be aged between 4 and 18 years, possess a certain level of cognitive ability to understand and execute simple rehabilitation training programs, have a history of previous joint bleeding confirmed through magnetic resonance imaging or ultrasound as synovitis or hemophilic arthropathy, exhibit symptoms of joint pain or restricted joint mobility, and be capable of completing a personalized rehabilitation therapy program lasting 12 weeks as planned. Participants were required to have no evidence of active bleeding on ultrasound and must have been free from any new bleeding episodes for at least four weeks before enrollment.

Participants with severe active bleeding, any bleeding within the past four weeks, or significant organ diseases such as heart, liver, or kidney disorders, or other serious abnormal conditions were excluded from this study (13).

### 2.2 Research grouping

Due to the strict inclusion criteria, resource limitations, and the rarity of cases, this study only included a total of 59 pediatric hemophilia patients who met the aforementioned inclusion criteria and received joint rehabilitation treatment at the Department of Rehabilitation Medicine, Affiliated Children's Hospital of Nanjing Medical University, from August 2017 to January 2025.

In the 12 weeks preceding the intervention, a significant increase in bleeding frequency was observed among participants, indicating that the prior prophylactic regimen may have been suboptimal. To address this concern, data on the weekly frequency of prophylactic treatments before the intervention were collected for each group. Based on recommendations from hematologists in the multidisciplinary team, a revised prophylactic factor VIII regimen was implemented: children with hemophilia A received prophylaxis three times per week, while those with hemophilia B received it twice per week, with each dose maintained at 20–25 IU/kg. During the intervention period, all 54 patients adhered strictly to the prescribed regimen, except for cases 8, 9, 28, 47, and 49, who received prophylaxis only once per week due to factors such as excessive body weight.

### 2.3 Ethics statement

This study strictly adhered to the principles outlined in the Helsinki Declaration and other relevant ethical guidelines. Prior to recruiting research participants, detailed information about the study, including its purpose, procedures, risks, and benefits, was provided to both the participants and their parents. A parent of each participant was required to sign an informed consent form after fully comprehending the study's contents before their participation could be considered. All participants' identities were kept strictly confidential and used solely for research purposes. We conducted a thorough risk assessment of potential risks associated with the study and implemented necessary measures to minimize any adverse effects to the greatest extent possible. Concurrently, we ensured that the research outcomes maximize benefits to both the participants and society. The study protocol was approved by the Medical Ethics Committee of the Affiliated Children's Hospital of Nanjing Medical University (Ethics Approval No: 202011088-1). All parents of the child participants were informed about the study details and results, and they provided their consent by signing the informed consent form. The committee was responsible for independently reviewing the ethical compliance of the study protocol and ensuring the protection of participants' rights throughout the research process.

### 2.4 Individualized rehabilitation therapy

**Figure 1:**
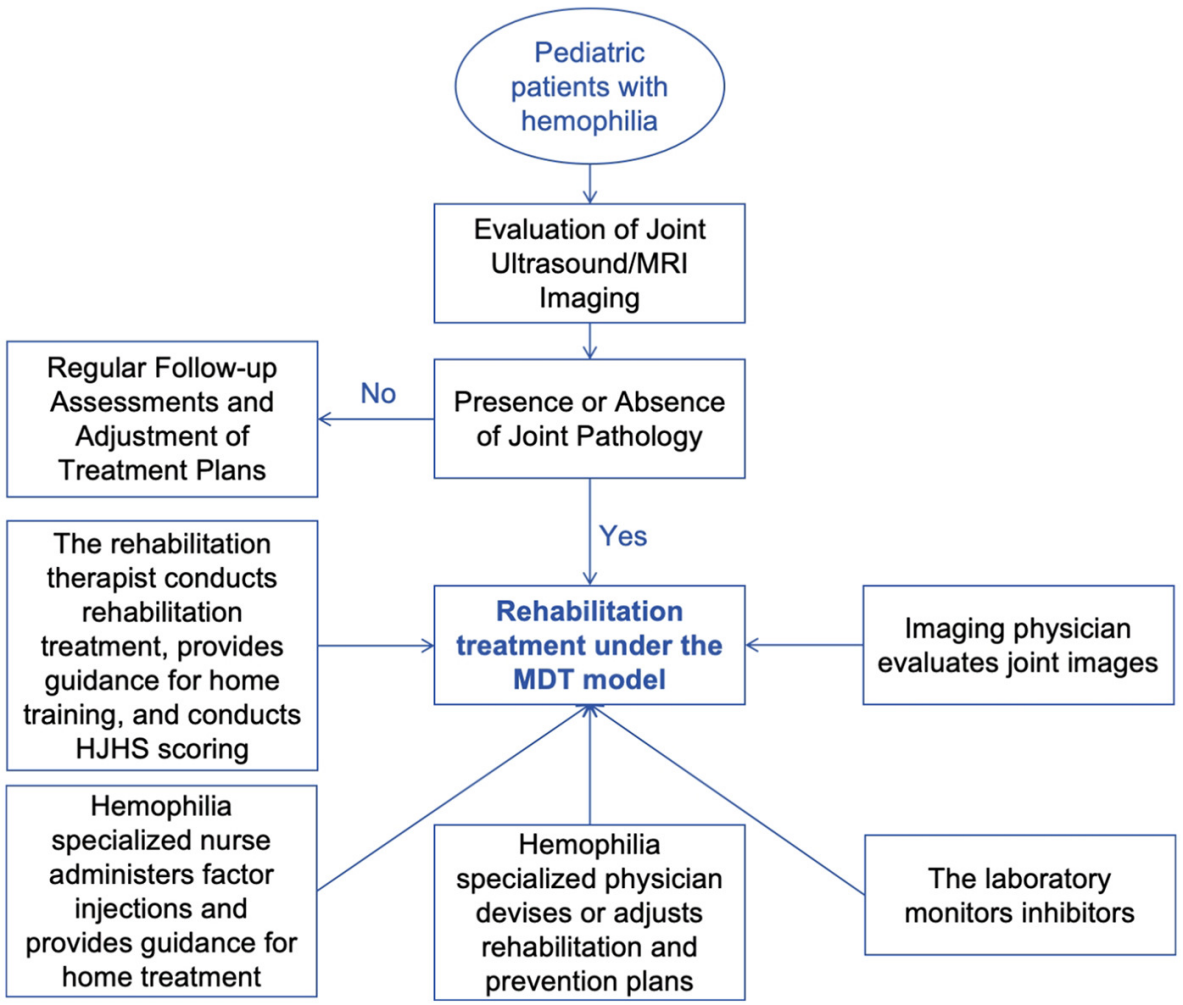
Individualized rehabilitation treatment under the multidisciplinary team model.

Following baseline assessments, a 12-week rehabilitation therapy program was initiated. This program included weekly hospital-based evaluation and treatment guidance sessions (60 min each) and five home-training sessions per week (45 min per session). Based on the individual assessment findings, personalized treatment techniques, medical assistive devices, home training content, and appropriate home-training durations were determined, taking into account the joint structure and functional status of each child. The exercise plan was designed to progress over time, with the intensity and duration of exercises adjusted according to each child's response to the therapy. The frequency of hospital-based therapy remained consistent at once per week, with each session lasting 60 min throughout the treatment cycle. Home-based therapy was conducted up to twice daily, with each session lasting 45 min. The intensity of home therapy began at 25 percent of the hospital therapy level, based on parameters such as the number of joint movements, weight-bearing, or resistance, and gradually increased to a maximum of 75 percent. Throughout the therapy process, it was essential to ensure that all joint and muscle activities remained pain-free. For preschool-aged children (seven years or younger), therapeutic content was incorporated into game-based activities or parent-child play to enhance their enthusiasm and participation in home therapy. Progress was closely monitored throughout the 12 weeks. Adherence to the home training program was monitored in several ways: parents submitted video check-ins via an online platform, rehabilitation therapists conducted regular online communications with families, and adherence was discussed during follow-up hospital visits. Although precise statistical data on adherence were not collected, parents reported that their children generally completed 30–50 min of exercise per session. The hematologist and rehabilitation physician worked together to develop a rehabilitation plan to ensure safety during the rehabilitation period. Any necessary medical support was provided in conjunction with the therapy (Figure 1).

#### 2.4.1 Individualized rehabilitation assessment measures

Rehabilitation therapy was performed on the same day as prophylactic factor administration. Prior to initiating treatment, children were evaluated using the HJHS 2.1 (14), which assesses joint limitations, swelling, and range of motion. Additional specialized assessments were also conducted to provide a more comprehensive understanding of joint and muscle function (Supplementary Materials).

#### 2.4.2 Individualized rehabilitation treatment measures

The individualized rehabilitation program focused on multiple aspects, including managing joint swelling; preventing muscle atrophy and promoting joint stability; improving restricted joint mobility; managing pain; enhancing muscle strength; and improving gait patterns. The goal was to provide comprehensive rehabilitation tailored to each patient's needs (Supplementary Materials).

#### 2.4.3 Online medical support

The MDT for hemophilia patients held online case discussions every two weeks. The treatment discussions were led by a rehabilitation specialist. During the 12-week intervention period, rehabilitation therapists conducted 12 scheduled online sessions to provide guidance and adjust the home training plans in real time based on photos and videos shared by the parents. These scheduled interventions were supplemented by unscheduled parent-initiated interactions to clarify instructions or make modifications as needed, ensuring the correct and safe implementation of the exercises.

#### 2.4.4 Home training guidance

Short- and long-term goals were established in the hospital based on each child's joint condition and activity level, and individualized home training programs were developed accordingly. The training followed the principles of safety and pain-free execution, with a gradual increase in joint range of motion, weight or resistance, and task difficulty. Body weight was prioritized as the primary source of resistance, supplemented with simple tools such as resistance bands and sandbags.

For children with restricted joint mobility, parents were instructed in safe stretching positions and appropriate traction techniques. Corrective sitting postures and weight-shifting exercises were also designed to address abnormal positioning. Additionally, simple sensory integration games were introduced to enhance balance, coordination, and proprioception. Psychological support was provided to help families cope with anxiety and emotional challenges at home (Supplementary Materials).

### 2.5 Data collection and evaluation methods

Data collection involved gathering the baseline information of the enrolled children, diagnostic details, and bleeding occurrences in the 12 weeks preceding rehabilitation as well as during the rehabilitation period.

The evaluation methods included the following (1): Hemophilic joint ultrasound examination, utilizing the Hemophilic Early Arthropathy Detection with UltraSound in China (HEAD-US-C) scale (15). The HEAD-US-C is an adaptation of the original HEAD-US protocol, tailored to better fit clinical practices and patient needs in China. This adaptation includes modifications in the scoring criteria for joint abnormalities and adjustments to ultrasound imaging techniques to accommodate different equipment and varying levels of operator expertise. All examiners for HEAD-US-C scoring were ultrasound physicians trained in hemophilic musculoskeletal ultrasound (2). Joint function assessment employing the HJHS 2.1 (14). The evaluators were rehabilitation therapists standardized through HJHS 2.1 training (3). Quality-of-life assessment using the Canadian Hemophilia Outcomes-Kids' Life Assessment Tool (CHO-KLAT) (16). Proxy reporting by parents was done for children aged 4–12 years, while those aged 12–18 years independently completed the assessment. During the assessment questionnaire process, communication between parents and children was prohibited. For items that were not fully understood, the researchers provided neutral, suggestive-free language explanations. Completed assessments were promptly collected to ensure questionnaire integrity.

### 2.6 Statistical methods

Data processing and analysis were conducted using IBM SPSS Statistics, version 25.0 (IBM Corp., Armonk, NY, USA). Descriptive statistics for continuous variables, were expressed as the mean ± standard deviation, while non-normally distributed data were presented as the median and interquartile range (IQR). To account for baseline differences, within-subject changes from baseline to post-treatment were calculated for key outcomes and analyzed using paired *t*-test or paired-sample Wilcoxon signed-rank tests. For comparing reductions in bleeding episodes between the prophylaxis and on-demand groups, independent samples *t*-tests or Mann–Whitney *U*-tests was conducted. The mean of these changes was reported to reflect the average treatment effect. This analysis assessed radiological changes, joint health, and quality of life in children with hemophilia before and after individualized rehabilitation therapy. Additionally, the safety of the therapy was evaluated by comparing bleeding episodes before and during the rehabilitation period. A significance level of *P* < 0.05 was considered statistically significant.

## 3 Results

### 3.1 Patient information and grouping

Fifty-nine children with hemophilia were enrolled, all males, with a median age of 7 years and 1 month, ranging from 4 to 17 years and 11 months. Among them, there were 52 cases of hemophilia A and 7 cases of hemophilia B; 16 cases were classified as moderate and 43 cases as severe. Prior to rehabilitation, 16 cases received on-demand treatment, while 43 cases received prophylactic treatment. There was a total of 70 affected joints, including 26 cases of unilateral ankle joint involvement, 15 cases of unilateral knee joint involvement, 7 case of unilateral elbow joint involvement, and 22 cases of involvement of two or more joints.

### 3.2 Changes in joint ultrasonography HEAD-US-C scores before and after rehabilitation therapy

**Figure 2:**
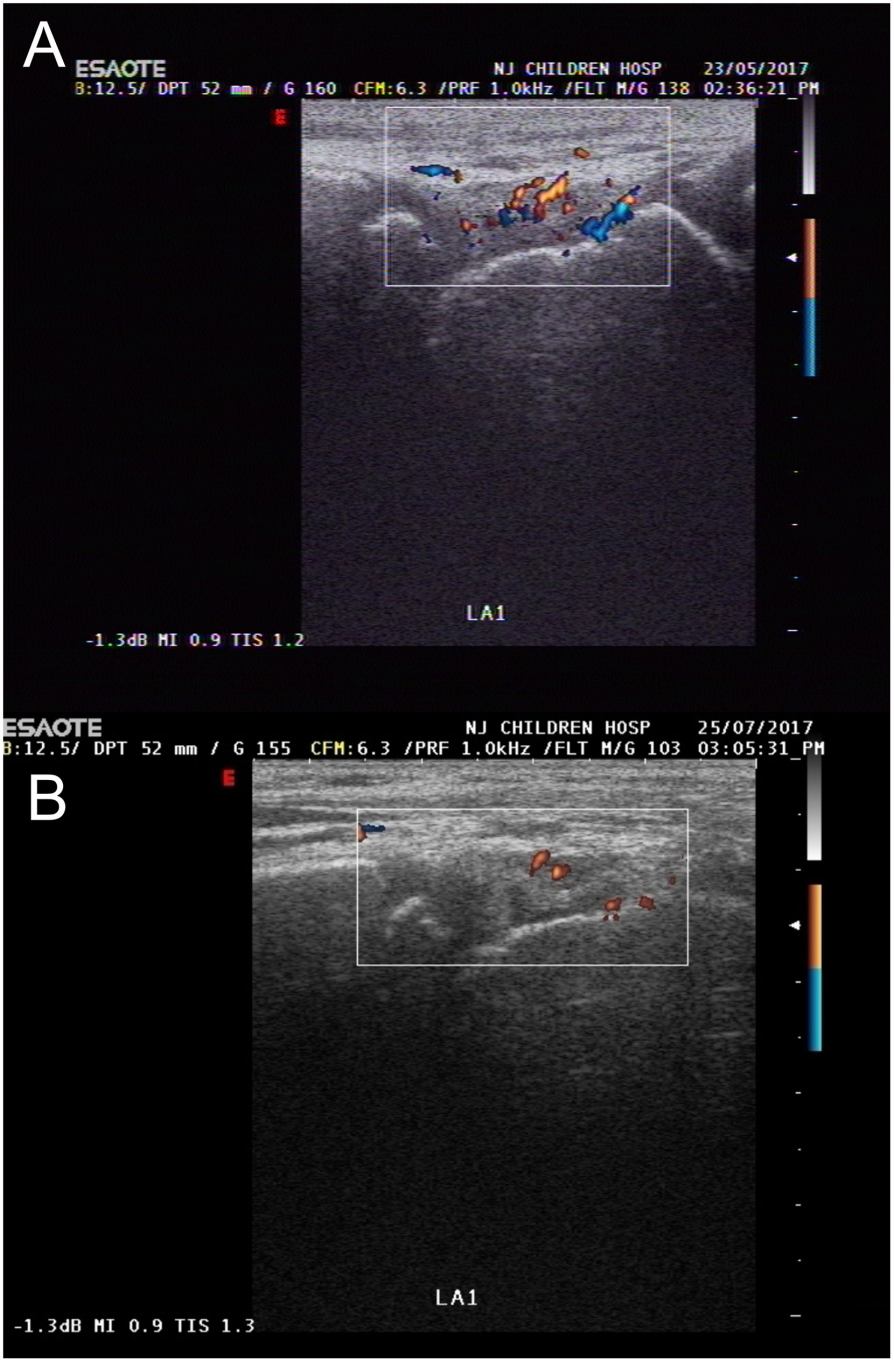
Comparison of left ankle joint condition before and after rehabilitation. **(A)** Before rehabilitation, synovial membrane thickening was observed in the left ankle joint, with the thickest part approximately 4 mm. No significant abnormalities in the joint cartilage or bone were observed. CDFI: Branching blood flow signals seen in the thickened synovial membrane. Left ankle joint synovial hyperplasia (2 points), synovial vascular proliferation (2 points). **(B)** After rehabilitation, synovial membrane thickening was observed in the left ankle joint, with the thickest part approximately 4 mm. No significant abnormalities in the joint cartilage or bone were observed. CDFI: Star-like blood flow signals seen in the thickened synovial membrane. Left ankle joint synovial hyperplasia (2 points), synovial vascular proliferation (1 point).

**Figure 3:**
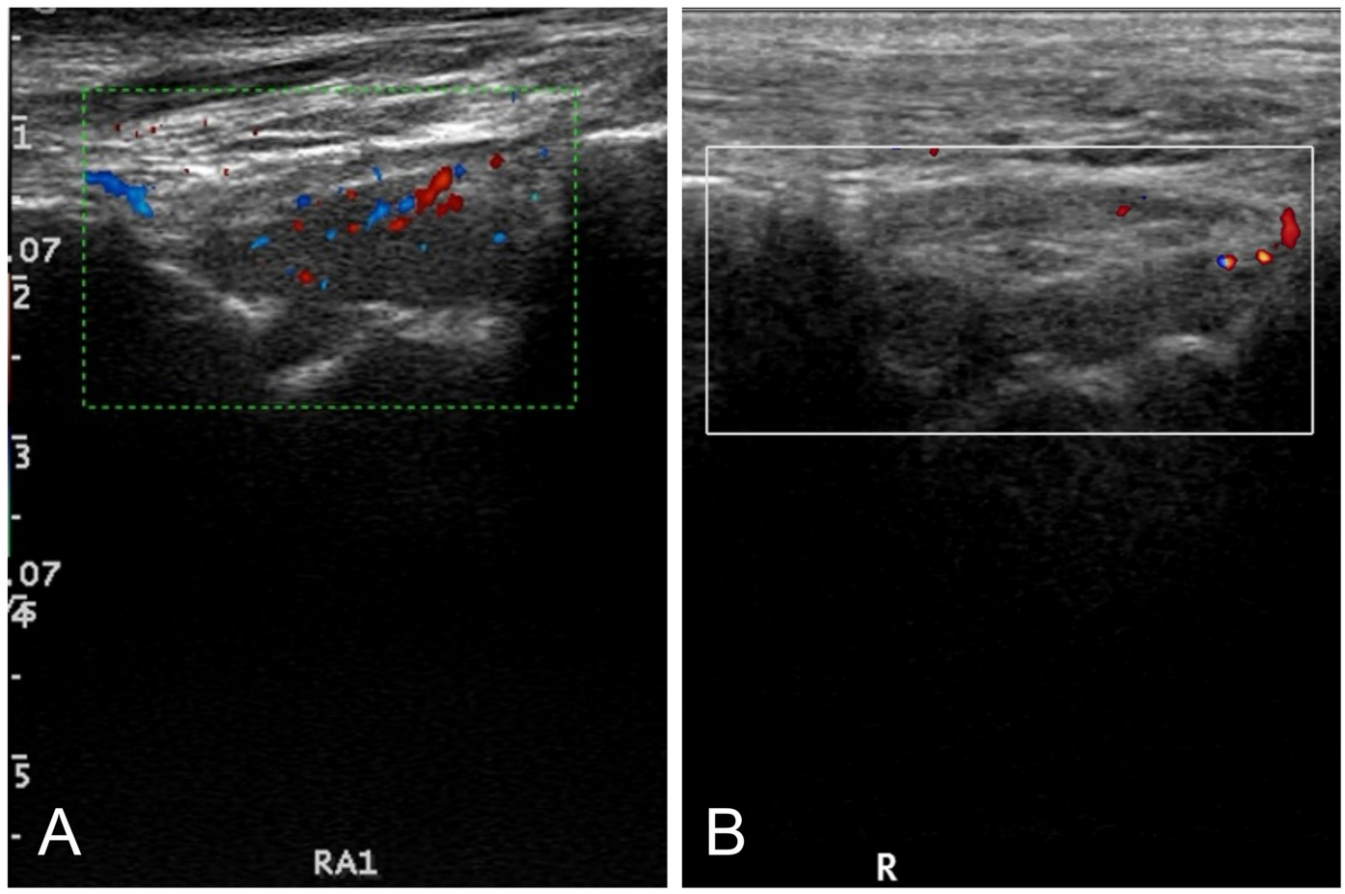
Ultrasonography of the right ankle joint before and after rehabilitation. **(A)** Before rehabilitation: synovial membrane thickening in the right ankle joint was observed, with the thickest part approximately 5.4 mm. Three-quarters of the joint cartilage was thinned, and the bone cortex was significantly irregular. CDFI: Linear blood flow signals were seen in the thickened synovial membrane. Right ankle joint synovial hyperplasia (2 points), synovial vascular proliferation (2 points), joint cartilage loss (3 points), bone destruction (2 points). **(B)** After rehabilitation: synovial membrane thickening in the right ankle joint was observed, with the thickest part approximately 5 mm. Three-quarters of the joint cartilage was thinned, and the bone cortex was significantly irregular. CDFI: Star-like blood flow signals were seen in the thickened synovial membrane. Right ankle joint synovial hyperplasia (2 points), synovial vascular proliferation (1 point), joint cartilage loss (3 points), bone destruction (2 points).

The duration of individualized rehabilitation therapy was 12 weeks. The median HEAD-US-C score before treatment was 5.00 (IQR: 2.00, 8.00); while after treatment, the median HEAD-US-C score was 1.00 (IQR: 0.00, 3.00). There was a significant decrease in the HEAD-US-C scores before and after rehabilitation (*Z* = −6.645, *P* < 0.001). To provide a clearer representation of the improvements observed in joint structure, a graph illustrating pre- and post-rehabilitation HEAD-US-C scores has been included in Figure 2. This figure highlights the significant decrease in joint abnormalities and allows for a more accessible understanding of the rehabilitation outcomes. A comparison of ultrasonography scores for joints with different degrees of damage revealed that early joint lesions such as synovial hyperplasia or joint effusion showed improvement after rehabilitation (Figure 2). However, severe joint lesions such as cartilage and bone destruction were irreversible after rehabilitation therapy (Figure 3).

### 3.3 Changes in hemophilia joint health scores (HJHS 2.1) before and after rehabilitation therapy

Before rehabilitation treatment, the median HJHS 2.1 score was 9.00 (IQR: 4.00, 15.00) points, which decreased to 2.00 (IQR: 1.00, 6.00) points after treatment. This indicates a significant improvement in joint health scores after rehabilitation treatment (*Z* = −6.632, *P* < 0.001), reflecting an enhancement in joint health. For a more intuitive display of the improvements in joint health, we have included a graph (Table 1) comparing the HJHS 2.1 scores before and after rehabilitation. This visual representation emphasizes the reduction in joint health score components such as muscle condition, range of motion, and swelling, providing a clearer picture of the rehabilitation's effectiveness. Further comparison of individual HJHS 2.1 scores revealed positive improvements in various aspects of joint health in children with hemophilia following rehabilitation treatment. For instance, the median muscle condition score decreased from 2.00 (IQR: 1.00, 4.00) points before treatment to 1.00 (IQR: 0.00, 2.00) points after treatment, indicating an increase in muscle strength and improvement in muscle atrophy (*Z* = −5.631, *P* < 0.001). Additionally, the median joint mobility score decreased from 2.00 (IQR: 0.00, 3.00) points before treatment to 0.00 (IQR: 0.00, 2.00) points after treatment, indicating a significant improvement in the range of motion of affected joints (*Z* = −5.261, *P* < 0.001). Furthermore, the bone crepitus score decreased from 1.00 (IQR: 0.00, 2.00) points before treatment to 0.00 (IQR: 0.00, 2.00) points after treatment, indicating a significant reduction in bone crepitus (*Z* = −4.794, *P* < 0.001). The gait score decreased from 2.00 (IQR: 1.00, 3.00) points before treatment to 0.00 (IQR: 0.00, 1.00) points after treatment, indicating a significant improvement in gait ability and movement skills (*Z* = −5.311, *P* < 0.001). The swelling score decreased from 2.00 (IQR: 1.00, 2.00) points before treatment to 0.00 (IQR: 0.00, 1.00) points after treatment, indicating a significant improvement in swelling after rehabilitation treatment (*Z* = −5.588, *P* < 0.001, Table 1).

**Table 1:** Changes in HJHS 2.1 scores before and after rehabilitation treatment^a^.

Category	Before rehabilitation	After rehabilitation	*Z*	*P*
HJHS 2.1	9.00 (4.00, 15.00)	2.00 (1.00, 6.00)	−6.632	<0.001
Muscle Strength and Atrophy	2.00 (1.00, 4.00)	1.00 (0.00, 2.00)	−5.631	<0.001
Flexion and Extension Sum	2.00 (0.00, 3.00)	0.00 (0.00, 2.00)	−5.261	<0.001
Crepitus	1.00 (0.00, 2.00)	0.00 (0.00, 1.00)	−4.794	<0.001
Gait	2.00 (0.00, 3.00)	0.00 (0.00, 1.00)	−5.311	<0.001
Swelling	2.00 (1.00, 2.00)	0.00 (0.00, 1.00)	−5.588	<0.001

^a^ Data are presented as the median and interquartile range. HJHS 2.1, Hemophilia Joint Health Score.

### 3.4 The quality-of-life scores before and after rehabilitation therapy

**Figure 4:**
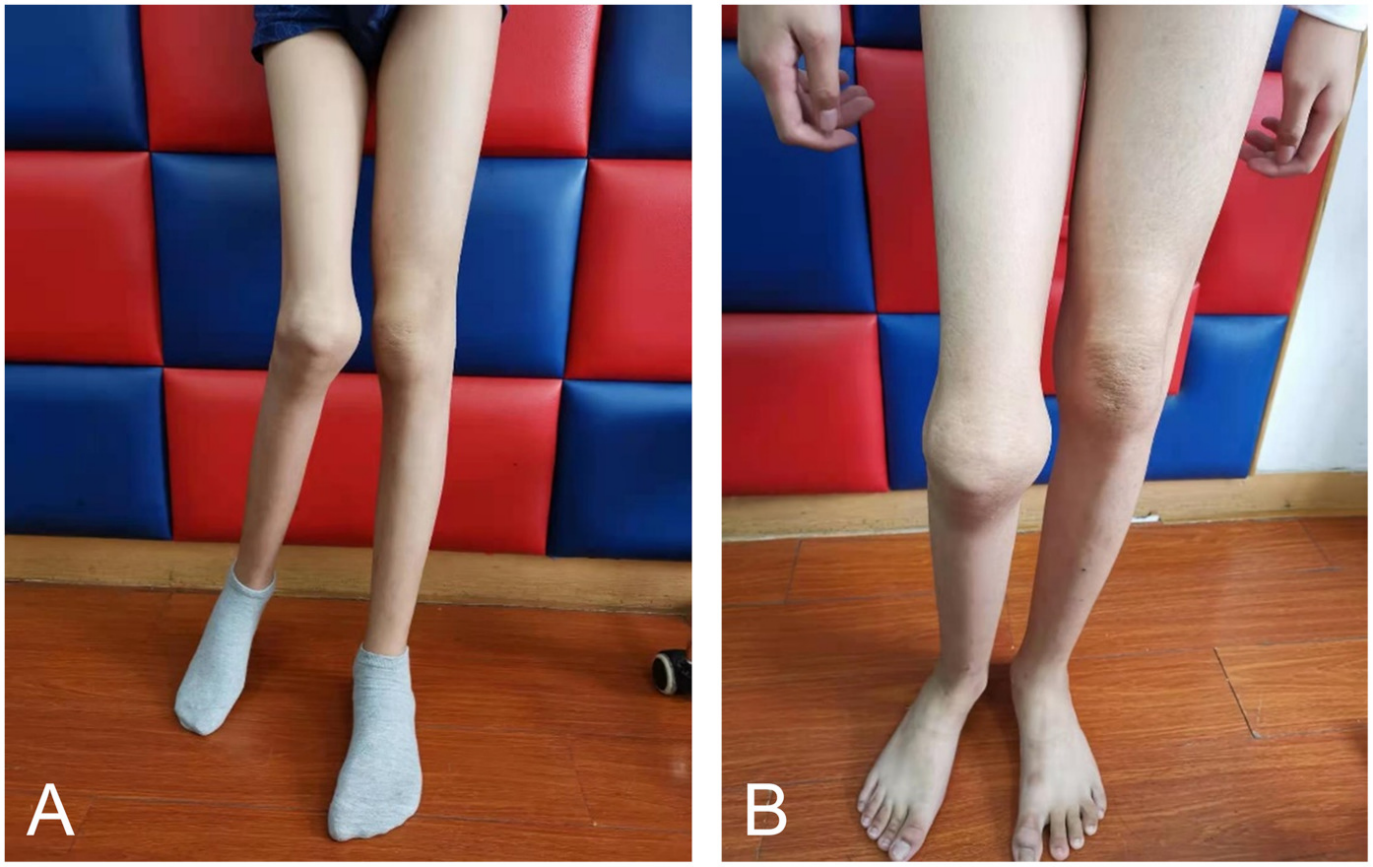
Comparison of the functional level before and after rehabilitation in a child with chronic hemophilic arthropathy. **(A)** Before rehabilitation: The right ankle could not bear weight, and the child was able to briefly stand against a wall but unable to stand independently. There was obvious asymmetry in the lower limb standing posture. **(B)** After rehabilitation: The right ankle could bear weight, and the child was able to stand independently with both legs. Improvements in the abnormal posture and asymmetry in the lower limb standing posture were observed.

During the 12-week individualized rehabilitation treatment, the median CHO-KLAT score before treatment was 71.51 (IQR: 60.46, 76.48) points, which increased to 79.15 (IQR: 773.45, 84.82) points after treatment. This signifies a significant improvement in quality of life after treatment (*Z* = −6.680, *P* < 0.001). Specific improvements were observed in physical functioning, emotional well-being, and social engagement, with enhanced mobility, reduced pain perception, and increased confidence in social situations. A graph depicting the CHO-KLAT scores before and after therapy visually illustrates these improvements (Supplementary Table S1). Additionally, rehabilitation led to significant gains in exercise function for children with delayed motor development (Figure 4), leading to further enhancement in the quality of life.

### 3.5 Changes in joint bleeding incidents before and during rehabilitation treatment

During the 12-week period before treatment, the median number of joint bleedings in the 59 cases was 3.00 (IQR: 2.00, 4.00) times. Throughout the 12 cycles of rehabilitation treatment, the median number of joint bleedings was 0.00 (IQR: 0.00, 1.00) times, indicating a decrease in the number of bleeding incidents during the treatment period compared to the same period before treatment (*Z* *=* −6.657, *P* *<* 0.001). Although the number of bleeding incidents was significantly reduced during the rehabilitation period, our analysis indicates no statistically significant difference in bleeding reduction between the prophylaxis and on-demand groups. The median reduction in bleeding episodes was 2.00 for the prophylaxis group and 2.00 for the on-demand group. Statistical tests (Mann–Whitney *U*-test *P* = 0.740) confirmed that this difference was not statistically significant (*P* > 0.05), suggesting that individualized rehabilitation effectively reduces the number of bleeding episodes regardless of the prerehabilitation prophylactic therapy. Future studies with larger sample sizes could provide further insights into these findings and validate the robustness of this effect across different patient groups (Supplementary Table S1).

### 3.6 Adherence to home rehabilitation

Adherence to the home rehabilitation program was generally high, as reported by parents, but certain challenges were noted during follow-up communications. While most children completed the recommended 30–50 min of exercise per session, five times per week, parents in two meetings reported that schoolwork occasionally prevented their children from completing the full training sessions. These time constraints highlighted the need for more practical and flexible training programs tailored to the schedules of school-aged children.

To address these challenges, future home rehabilitation plans could incorporate shorter, more frequent sessions that utilize fragmented time available for school-aged children. Additionally, safe, interactive activities could serve as partial substitutes for formal training to ensure consistency while maintaining engagement. These adjustments could improve adherence while reducing the burden on families, ensuring that children receive the full benefit of rehabilitation therapy. Although precise adherence data were not tracked quantitatively in this study, these proposed solutions aim to enhance feasibility and adherence in future implementations.

## 4 Discussion

Joint muscle bleeding is common in children with hemophilia due to inadequate treatment, leading to recurrent bleeds and joint damage, particularly in weight-bearing joints like the knees and ankles (17). These lesions significantly impact the quality of life of children with hemophilia and may have long-term consequences into adulthood (18).

Our study assessed the efficacy of personalized rehabilitation therapy in alleviating joint swelling and reversing synovial hypertrophy and effusion in affected joints. The rehabilitation plan focused on replicable components like muscle strength, swelling, and joint mobility, ensuring consistency within an individualized framework. Additionally, home exercise guidance was provided based on these core principles, making the rehabilitation plan reproducible in similar clinical settings.

All children with hemophilia included in this study experienced spontaneous single- or multiple-joint bleeding within one month prior to enrollment due to a lack of standardized joint health management. This led to joint dysfunction and an impaired ability to perform daily activities, necessitating systematic rehabilitation treatment. We found that early rehabilitation is crucial for joint recovery in children with hemophilia, as it can improve overall joint imaging structures and restore joint movement patterns affected by the lesions (19). Despite some cases showing no improvement in imaging structure post-rehabilitation, likely due to irreversible cartilage or bone damage from repeated joint bleeds, significant improvements were observed in the muscle condition and joint motion range (20).

This study aimed to compare individual outcomes before and after rehabilitation therapy, providing preliminary insights into the effectiveness of personalized rehabilitation in improving joint health in children with hemophilia. However, this design may lead to two issues: (1) Ambiguous attribution of effects: for example, improvements in joint range of motion may result from the combined effect of natural recovery after reduced bleeding due to prophylaxis and rehabilitation training, rather than from rehabilitation alone. (2) Limited extrapolation of conclusions: the study can only demonstrate the overall effectiveness of the combined intervention, but cannot answer questions such as whether rehabilitation therapy can be safely implemented in patients without adequate prophylaxis, or whether rehabilitation alone has clinical value.

It is important to note that the current dataset lacks complete records on treatment adherence and dosage adjustments, which may limit the depth of analysis regarding the association between prophylactic treatment and rehabilitation outcomes.

While all patients received some level of prophylaxis during the study, specific regimens varied; 16 of the 59 patients transitioned from on-demand therapy to at least one prophylactic injection per week during rehabilitation. Prophylaxis alone is well-established in reducing bleeding episodes and supporting joint stability, likely contributing to the observed improvements. Future studies should address these limitations by systematically collecting precise data on prophylaxis regimens and factor VIII consumption, as well as incorporating a control group with comparable prophylaxis levels but no rehabilitation, in order to clarify the independent effects and appropriate scope of rehabilitation therapy. This would provide stronger evidence for optimized resource allocation and precision medicine. Furthermore, multivariable statistical analyses will be necessary to isolate the specific effects of rehabilitation from other influencing factors, such as prophylaxis adjustments, baseline joint health, and age.

Despite these limitations, this study offers valuable baseline data for larger-scale investigations and highlights the potential of personalized rehabilitation to improve joint health and quality of life in pediatric hemophilia patients. Under standardized prophylactic treatment, personalized rehabilitation may serve as a complementary approach to enhance joint function. However, this study focused exclusively on male children with hemophilia undergoing rehabilitation, excluding female patients, different age groups, and varied treatment settings, which severely limits the generalizability of the findings. The results may not be applicable to broader populations. Future studies will aim to expand sample diversity and conduct multicenter, stratified research to enhance the external validity of the findings.

Analysis of the HJHS 2.1 scores revealed improvements in several joint health parameters, including reduced joint pain, improved range of motion, and decreased swelling and crepitus (21). While the improvement in HJHS scores was statistically significant, clinical studies indicate that any reduction in the score reflects therapeutic efficacy, highlighting the practical significance of our findings for patient outcomes. While the HJHS does not specifically assess the muscle condition, other assessments performed in conjunction with the HJHS measure muscle atrophy, strength, and tone, among other parameters. Individualized rehabilitation addresses these muscle-related issues, improving muscle tone and strength, correcting body alignment, and reducing joint creases and swelling with assistive devices. Therefore, personalized rehabilitation therapy can effectively promote the improvement and recovery of early joint lesions in children with hemophilia to some extent (22).

Our research highlights the positive impact of personalized rehabilitation therapy on the quality of life of children with hemophilia. We observed significant improvements in their willingness to engage in activities, emotional well-being, and motor function, all contributing to an enhanced quality of life.

In routine management, children with hemophilia typically receive on-demand treatment, with rehabilitation services being sought only when joint issues severely affect function (23). The focus is on the safety and efficacy of rehabilitation therapy without reference to specific prophylactic adjustments. Rehabilitation processes face challenges due to the lack of specialized communication platforms between departments. This results in delayed access to important patient information for rehabilitation therapists, including imaging data, medication details, and patient compliance. Moreover, the inherent bleeding tendency in hemophilia patients leads to conservative approaches during assessment and treatment, potentially resulting in the inadequate resolution of acute symptoms and an increased risk of chronic multi-joint problems (24).

Our study demonstrates the effectiveness of a multidisciplinary collaborative care model in managing hemophilia-related joint disease in children. Through our case series, healthcare professionals from various disciplines not only fulfilled their departmental roles but also established a collaborative model centered around children with hemophilia. This included hemophilia specialists devising prophylactic treatment plans to ensure safety during rehabilitation, the imaging department conducting joint ultrasound examinations for monitoring, and hematology nurses providing home safety education and collecting quality-of-life ratings. These collaborative efforts effectively met the clinical needs of children with hemophilia and their families.

Expanding on this groundwork, rehabilitation therapists implemented a personalized rehabilitation plan based on HJHS2.1 scores. Though HJHS 2.1 is not typically used for treatment management, its detailed assessment of joint restrictions, muscle atrophy, and movement limitations provided valuable data for tailoring exercises to each patient's specific deficits, focusing on range of motion, muscle strengthening, and swelling reduction. This enabled individualized rehabilitation plans aligned with each patient's unique joint health profile. This approach not only utilized in-house rehabilitation but also incorporated online communication with children and their families via a user-friendly network platform, allowing for prompt adjustments to home training programs and providing psychological support (25). However, exercise poses a bleeding risk in hemophilia patients, necessitating rehabilitation therapy to minimize iatrogenic damage or bleeding risk (3). Thus, hemophilia specialists must adjust or establish prophylactic treatment plans to ensure smooth rehabilitation based on the conditions of patients. Especially for pediatric patients, rehabilitation plans should consider their unique characteristics and emphasize education on daily protection to minimize interference from acute bleeding. Incorporating warm-up activities and post-session stretching further enhanced treatment safety, thus contributing to overall improvements in the cardiopulmonary function of the children (26).

In this study, children undergoing personalized rehabilitation therapy experienced a significant reduction in the total number of bleeding episodes during the treatment period. During the intervention, all 59 patients received prophylaxis, with prior treatment regimens varying between the groups. Before the intervention, 43 patients were already on prophylaxis, and 16 patients were managed with on-demand therapy. However, we did not systematically collect detailed data on the specific prophylactic regimens (IU/kg/week) or factor VIII consumption (IU/kg) for either group before or during the intervention. While adjustments to prophylaxis during the intervention were reported to be minimal and aligned with standard care protocols, this lack of detailed data limits our ability to fully evaluate the contribution of prophylaxis modifications to the observed outcomes. In future studies, we will prioritize the collection of detailed prophylactic treatment data to enable a more comprehensive analysis of treatment outcomes.

This study's limitations include the lack of a control group, which restricts direct comparison with other therapies. Future studies could benefit from a control group and tracking of the specific contributions of rehabilitation and prophylaxis. Another limitation is the absence of quantitative monitoring for adherence to home rehabilitation. The variability in adherence may have introduced a confounding factor in interpreting the results, as differences in patient engagement could influence outcomes. While parents reported high adherence, structured tracking could reduce variability in interpreting adherence-related outcomes. In future research, we will implement a formal and standardized adherence monitoring system, such as electronic monitoring devices or patient diaries, to ensure accurate and consistent data collection and improve the reliability of the study findings.

Only a few cases of subcutaneous bleeding or bruising were reported, all of which were associated with external trauma or accidental collisions, likely related to the natural activity levels of young male children. Importantly, these episodes did not occur on the days of in-house rehabilitation sessions or during home training periods. No adverse events were recorded in relation to the rehabilitation program itself.

It is important to note that while the adjustments made to the prophylaxis regimens during the study likely contributed to the improvements observed in joint health and quality of life, the rehabilitation therapy itself played a crucial role. This study concludes that personalized rehabilitation therapy effectively enhances joint health and quality of life in pediatric hemophilia patients. These results lay the groundwork for future research to further explore long-term outcomes and refine rehabilitation strategies. Integrating this personalized rehabilitation model into routine clinical practice may reduce treatment time and costs, streamline hospital-home care, and provide comprehensive, individualized support for children with hemophilia. Additionally, it will minimize disruptions to daily life and academics for school-age children due to rehabilitation therapy.

## 5 Conclusion

In conclusion, this study offers preliminary insights into personalized joint rehabilitation therapy for children with hemophilia-related joint disease. Through pre- and post-rehabilitation comparisons, personalized therapy was found to significantly improve the joint structure, function, and quality of life in children with hemophilia-related joint lesions. In exploring rehabilitation models, we sought to extend medical services into the home setting. With the support of future evidence-based research, this approach may be further optimized to enhance treatment adherence among both patients and caregivers. Effective implementation in clinical practice requires close collaboration within a multidisciplinary team. Coordinated efforts among departments such as hematology, rehabilitation, and nursing are essential to improving intervention efficiency. The integration of such services has the potential to promote an innovative “prevention–rehabilitation–home management” framework, providing comprehensive and continuous care for children with hemophilia and supporting joint function recovery across the full treatment cycle (27). However, there are several limitations in this study. First, it adopted a retrospective design and involved a limited population scope, focusing solely on children who underwent rehabilitation treatment as well as regular reviews and evaluations. Additionally, the sample consisted only of male pediatric hemophilia patients, which may limit the generalizability of the findings to other hemophilia populations. This approach prevented comparison with children having joint lesions who did not receive rehabilitation treatment. Future studies could incorporate a more rigorous tracking method for home training and extend the follow-up period to enhance the consistency of home training and assess the long-term effects of rehabilitation. Second, considering the developmental stage of children and the influence of factors such as different age groups and varying degrees of joint lesions on the efficacy of rehabilitation treatment, more clinical data accumulation and analysis are necessary. To address these limitations, future research should entail multicenter studies with expanded sample sizes, enhanced treatment and assessment compliance among children with hemophilia, prolonged observation periods, and the establishment of control groups or cohorts comprising children and adults. This comprehensive approach will facilitate a more thorough evaluation of the effects of rehabilitation treatment under the MDT model on children at different developmental stages and with varying severity of joint lesions. Ultimately, this will provide an objective basis for the establishment of a scientific and standardized rehabilitation model for hemophilia-related joint diseases.

## Data Availability

The raw data supporting the conclusions of this article will be made available by the authors, without undue reservation.
