# Supplementary material for: The effect of prophylactic FVIII infusion combined with personalized rehabilitation on joint health and quality of life in children with hemophilia
Source: Front Pediatr. 2025 Jul 10;13:1578617. doi: 10.3389/fped.2025.1578617 (PMC12287007; doi:10.3389/fped.2025.1578617)
Supplement: Supplementary file 1 [file Datasheet1.doc]

**Supplementary Material**

*Detailed measures for individualized rehabilitation assessment*

In-hospital rehabilitation therapy was conducted on the same day as prophylactic clotting factor infusion. On treatment days, participants wore loose-fitting separates to facilitate assessment and exercise training. The therapy adhered to the principles of progressive intensity and pain-free movement, aiming to restore the full range of joint motion. Prior to treatment initiation, the children were assessed using the HJHS 2.1 , which evaluates joint restrictions, swelling, and range of motion but does not specifically assess muscle functionality. To address this limitation, additional assessments were conducted to provide a more comprehensive understanding of muscle functionality. These included measuring muscle tone using the Ashworth grading scale to assess spasticity levels, observing for synergistic movements in adjacent joints during joint activity, and analyzing body posture in a seated position. While these supplementary assessments offered valuable insights, they were not included in the final analysis as they did not significantly impact the outcomes. Using the combined results of the HJHS 2.1 and supplementary assessments, treatment plans were formulated based on the guidelines for osteoarthritis rehabilitation treatment and Kathy Mulder’s guidance on hemophilia exercise . This individualized approach ensured that the rehabilitation program addressed each child’s specific deficits and functional needs.

*Specific measures for individualized rehabilitation therapy*

(1) Manage swelling. The main objective was to expedite swelling absorption and to reduce its duration. Common methods included the following: i. utilizing pain-free range-of-motion exercises combined with centripetal massage for swollen joints; ii. employing cryotherapy during the acute phase to decrease swelling and applying electrotherapy or ultrasound in the post-acute phase to enhance blood circulation and to facilitate swelling absorption; iii. applying compression bandages post-treatment, gradually decreasing the pressure from the limb’s distal end to the proximal end.

(2) Treat muscle atrophy and crepitus. To inhibit muscle atrophy, enhance joint stability, and reduce crepitus, common methods included the following: i. the use of weight-bearing or resistance exercises for muscle atrophy; ii. the use of Rood techniques to enhance the sensory input of atrophied muscles, a method grounded in the principle of neuroplasticity, where sensory stimulation is used to facilitate motor responses in weak or atrophied muscles; iii. the application of joint compression techniques to promote coordinated muscle contraction and to stabilize the joint, which is particularly effective for providing proprioceptive feedback that enhances motor control and joint stability in hemophilia patients. The choice of these techniques was based on their efficacy in improving muscle function and joint stability in pediatric rehabilitation settings.

(3) Treat restricted joint mobility (decreased flexion or extension). To improve joint mobility and to restore its maximum range of motion, common methods included the following: i. passive and active range-of-motion exercises within the pain-free range of motion of the restricted joint; ii. utilizing specific positions, continuous passive motion machines, or manual stretching of key muscles; iii. strength training of antagonist muscles.

(4) Treat joint pain. To alleviate pain in children through physical therapy and psychological interventions, common methods included the following: i. various physical modalities (e.g., electrotherapy, hydrotherapy, and laser therapy) to alleviate joint and muscle pain; ii. providing guidance and support for psychological issues caused by pain to improve the psychological state of the children and to reduce their focus on pain.

(5) Improve muscle strength. For children with inadequate muscle strength around the joint and poor joint stability, resistance training, gravity-resisted, or weight-reduced muscle strength training models were adopted according to different strength levels. Common exercises included the following: i. kneeling push-ups; ii. single-leg squats with a slow descent; and iii. wall squats.

(6) Improve walking techniques (e.g., walking, stair climbing, running, and single-leg hopping). To correct abnormal gait patterns and body alignment as well as to establish safe running and hopping patterns, common methods included the following: i. partial weight-bearing walking systems; ii. obstacle-crossing training; iii. 10-meter deceleration and return running training; and iv. alternating single and double-leg hopping exercises on a children’s trampoline.

(7) Other treatments. For children often accompanied by balance and coordination impairments, common treatment methods included the following: i. dynamic rowing exercises (sitting on a Bobath ball, alternating movements resembling rowing with both hands); ii. dynamic ball-catching exercises (sitting on a Bobath ball or standing on a balance pad, tossing and catching balls with the therapist); iii. point support (hands and knees diagonal support position, alternating support). Health education was provided to children with hemophilia and their parents. The suitable daily exercise content was selected based on the child’s joint level and age to reduce prolonged stair climbing and mountain climbing, avoid vigorous physical activities, reduce obesity in overweight children, and alleviate pain during lower limb joint loading.

Note: For the treatment of early joint lesions such as synovial hyperplasia or joint effusion (Grade I–II), the main goal was inflammation control. We employed physiotherapy devices such as shortwave diathermy and cryotherapy machines. Concurrently, rehabilitation focused on restoring joint range of motion and implementing muscle protection training. For instance, passive flexion-extension training of the knee in the supine position (0–90°) was conducted with 10 repetitions per session, combined with wall-slide exercises (sliding the heel along a towel to increase flexion angle), and quadriceps isometric contractions (leg extended, muscle tensed and held for 5 seconds, 10 reps/set, 3 sets daily).

In cases of severe joint damage such as cartilage erosion and bony destruction (Grade III–IV), the therapeutic objective shifted toward enhancing muscle strength and delaying structural deterioration. Joint mobilization techniques were appropriately applied. For severe contractures, progressive orthotic correction was used: during the day, adjustable hinged orthoses were worn, while static orthoses set to the maximum tolerable angle were used at night. In this stage, special attention was paid to pain management. For example, in cases of elbow bone destruction accompanied by lateral epicondylitis, resistance training was suspended and pain management was prioritized.

For all children undergoing rehabilitation involving the knee or ankle, gait analysis and guidance were provided to reduce abnormal weight-bearing patterns.

*Online medical support*

The MDT for hemophilia patients, including specialists from the hematology, rehabilitation, imaging, and laboratory departments, held online case discussions every two weeks using Tencent Meeting software, with each session lasting 30 min. Tencent Meeting is one of the most widely used platforms in China; however, we took additional measures to ensure privacy and confidentiality. For every session, a unique login password was set and shared only with professionals and parents after confirming the meeting time. To enhance security, the password was sent just before the meeting began, minimizing the risk of unauthorized access.

The team, led by a specialist in rehabilitation, also used the WeChat platform as a communication channel for treatment discussions. While WeChat employs encryption to protect messages, additional efforts were made to secure sensitive medical data. Rehabilitation therapists conducted 12 scheduled online interventions during the 12-week intervention period to provide guidance and promptly adjust home training programs based on the images and videos shared by parents. These scheduled sessions were supplemented by additional unscheduled interactions initiated by parents for clarification or modifications as needed, ensuring the correct and safe implementation of exercises.

Although the specific number of psychological support interventions was not formally recorded, counselors provided support as required during these online communications with parents of children experiencing emotional challenges. This flexible approach ensured that both technical guidance and emotional support were accessible throughout the intervention period, addressing the needs of both children and their families.

*Home training guidance*

At the hospital, short-term and long-term goals were set based on the child’s joint condition and activity level, and the personalized home training content was developed. The training content was conducted under the premise of safety and painlessness and followed a progressive sequence: gradually increasing joint range of motion, weight or resistance from light to moderate, and training difficulty from easy to challenging. Prior to training, short warm-up activities were performed to avoid the affected joints. Since most families lack complex professional training equipment, body weight was preferred as the ideal resistance source, followed by the use of simple training aids such as elastic bands and sandbags, according to specific circumstances, for training the strength and endurance of key muscles. For children with restricted joint mobility, their parents were instructed on safe stretching positions and appropriate traction force input, mastering simple stretching techniques. Abnormal body postures were corrected, and different sitting and standing positions as well as weight-shifting exercises were designed, with the necessary use of home rehabilitation aids if needed. The balance, coordination, and proprioceptive abilities of the children were strengthened through the design of simple sensory integration games, allowing them to complete such training during game activities, thereby enhancing their enthusiasm. During home training, the parents were not allowed to arbitrarily change the training intensity or content. Adherence to the home-based exercises was monitored informally but not rigorously tracked. In addition, during the home training guidance, we provided preemptive psychological support to address any anxiety, tension, or other emotional challenges that parents or children might experience at home. Individual children may have experienced adverse reactions such as excessive fatigue or mild sleep disturbances after training, which could be resolved through timely consultation with online experts or scheduling offline appointments for diagnosis and treatment.

**References**

1. Feldman BM, Funk SM, Bergstrom BM, Zourikian N, Hilliard P, van der Net J, et al. Validation of a new pediatric joint scoring system from the International Hemophilia Prophylaxis Study Group: validity of the hemophilia joint health score. *Arthritis Care Res (Hoboken)*. (2011) 63:223-30. doi: 10.1002/acr.20353

2. Chinese Orthopedic Association, Joint Surgery Group of Chinese Medical Association. Diagnosis and Treatment Guidelines for Osteoarthritis (2018 Edition). *Chin J Orthop*. (2018) 38:705-15. doi: 10.3760/cma.j.issn.0253-2352.2018.12.001

3. Zheng GX, Yang Y, Tang JS, Liang Y. Expert consensus on rehabilitation treatment for osteoarthritis. *Chin J Phys Med Rehabil*. (2012) 34:951-53.

4. Strike K, Mulder K, Michael R. Exercise for haemophilia. *Cochrane Database Syst Rev*. (2016) 12:Cd011180. doi: 10.1002/14651858.CD011180.pub2
