# Supplementary material for: The effect of prophylactic FVIII infusion combined with personalized rehabilitation on joint health and quality of life in children with hemophilia
Source: Front Pediatr. 2025 Jul 10;13:1578617. doi: 10.3389/fped.2025.1578617 (PMC12287007; doi:10.3389/fped.2025.1578617)
Supplement: Supplementary file 2 [file Table1.doc]

**Table S1.** Clinical Data of 59 Pediatric Cases with Individualized Rehabilitation Treatment under the MDT Model

| Case | Age (years) | Type of Hemophilia | Severity | Pre-Rehabilitation Replacement Therapy | HJHS 2.1 Pre-Rehabilitation Score | HJHS 2.1 Post-Rehabilitation Score | CHO-KLAT Pre-Rehabilitation Score | CHO-KLAT Post-Rehabilitation Score | Bleeding Episodes Pre-Rehabilitation (12 weeks) | Bleeding Episodes During Rehabilitation (12 weeks) |
| --- | --- | --- | --- | --- | --- | --- | --- | --- | --- | --- |
| 1 | 4.00 | HA | Severe | Prophylaxis | 12 | 1 | 66.41 | 80.01 | 4 | 0 |
| 2 | 6.00 | HA | Severe | Prophylaxis | 11 | 5 | 62.56 | 72.13 | 3 | 1 |
| 3 | 5.00 | HA | Severe | Prophylaxis | 12 | 2 | 62.9 | 71.77 | 1 | 0 |
| 4 | 8.00 | HA | Severe | On-Demand | 9 | 3 | 66.73 | 75.89 | 3 | 0 |
| 5 | 12.00 | HA | Moderate | On-Demand | 26 | 6 | 54.78 | 78.46 | 3 | 1 |
| 6 | 11.00 | HB | Severe | On-Demand | 16 | 8 | 60.89 | 83.11 | 1 | 0 |
| 7 | 4.50 | HA | Severe | Prophylaxis | 11 | 3 | 47.71 | 68.45 | 2 | 1 |
| 8 | 13.50 | HA | Severe | On-Demand | 42 | 31 | 31.45 | 53.56 | 2 | 2 |
| 9 | 10.17 | HA | Severe | On-Demand | 22 | 6 | 49.68 | 77.36 | 2 | 1 |
| 10 | 4.25 | HA | Severe | Prophylaxis | 15 | 8 | 46.59 | 58.79 | 3 | 2 |
| 11 | 8.58 | HA | Severe | Prophylaxis | 10 | 3 | 61.57 | 84.79 | 3 | 1 |
| 12 | 9.00 | HA | Moderate | Prophylaxis | 13 | 2 | 71.93 | 88.45 | 2 | 0 |
| 13 | 5.17 | HB | Severe | Prophylaxis | 7 | 5 | 69.68 | 74.56 | 3 | 0 |
| 14 | 5.17 | HA | Moderate | On-Demand | 17 | 16 | 57.32 | 65.45 | 4 | 2 |
| 15 | 5.00 | HB | Moderate | Prophylaxis | 4 | 1 | 76.67 | 85.35 | 3 | 0 |
| 16 | 7.00 | HA | Moderate | On-Demand | 10 | 5 | 60.4 | 76.6 | 2 | 0 |
| 17 | 6.42 | HA | Severe | Prophylaxis | 4 | 4 | 75.51 | 84.82 | 4 | 2 |
| 18 | 5.00 | HA | Severe | Prophylaxis | 22 | 8 | 53.63 | 70.55 | 5 | 0 |
| 19 | 4.58 | HA | Moderate | Prophylaxis | 7 | 0 | 71.51 | 89.4 | 3 | 0 |
| 20 | 14.92 | HA | Severe | Prophylaxis | 8 | 7 | 75.63 | 78.45 | 4 | 1 |
| 21 | 7.92 | HA | Severe | Prophylaxis | 23 | 16 | 55.21 | 67.84 | 4 | 1 |
| 22 | 4.25 | HA | Severe | Prophylaxis | 2 | 0 | 81.24 | 88.11 | 2 | 0 |
| 23 | 12.00 | HA | Moderate | Prophylaxis | 12 | 5 | 76.48 | 85.27 | 3 | 0 |
| 24 | 6.67 | HA | Moderate | On-Demand | 19 | 14 | 58.65 | 64.51 | 4 | 1 |
| 25 | 8.00 | HA | Severe | Prophylaxis | 19 | 10 | 49.53 | 65.37 | 4 | 2 |
| 26 | 13.25 | HA | Severe | Prophylaxis | 7 | 5 | 72.45 | 78.96 | 2 | 1 |
| 27 | 4.33 | HA | Severe | Prophylaxis | 9 | 1 | 62.35 | 80.16 | 2 | 0 |
| 28 | 8.00 | HA | Moderate | On-Demand | 34 | 27 | 34.17 | 50.57 | 6 | 2 |
| 29 | 9.00 | HA | Severe | Prophylaxis | 6 | 5 | 72.59 | 77.76 | 2 | 0 |
| 30 | 10.00 | HA | Moderate | On-Demand | 6 | 0 | 75.43 | 82.48 | 2 | 0 |
| 31 | 6.00 | HA | Severe | Prophylaxis | 1 | 0 | 81.57 | 85.43 | 1 | 0 |
| 32 | 4.25 | HA | Severe | Prophylaxis | 7 | 1 | 70.14 | 79.90 | 2 | 0 |
| 33 | 5.25 | HA | Moderate | Prophylaxis | 7 | 1 | 74.55 | 80.47 | 3 | 0 |
| 34 | 5.08 | HA | Severe | Prophylaxis | 2 | 0 | 79.89 | 83.25 | 1 | 0 |
| 35 | 7.00 | HB | Severe | Prophylaxis | 10 | 7 | 68.73 | 72.65 | 3 | 1 |
| 36 | 7.08 | HA | Severe | Prophylaxis | 4 | 0 | 76.47 | 86.63 | 2 | 0 |
| 37 | 5.00 | HA | Severe | Prophylaxis | 2 | 1 | 86.46 | 88.35 | 2 | 0 |
| 38 | 15.83 | HB | Severe | Prophylaxis | 23 | 15 | 56.47 | 64.11 | 5 | 2 |
| 39 | 6.75 | HA | Severe | Prophylaxis | 1 | 0 | 85.46 | 89.27 | 1 | 0 |
| 40 | 4.00 | HA | Severe | Prophylaxis | 2 | 1 | 85.56 | 88.42 | 2 | 0 |
| 41 | 5.00 | HB | Severe | Prophylaxis | 3 | 0 | 80.31 | 85.35 | 3 | 0 |
| 42 | 7.50 | HA | Moderate | On-Demand | 13 | 3 | 63.74 | 73.45 | 4 | 1 |
| 43 | 8.25 | HA | Severe | Prophylaxis | 6 | 4 | 74.43 | 78.56 | 2 | 1 |
| 44 | 15.00 | HA | Severe | On-Demand | 19 | 7 | 58.32 | 75.21 | 4 | 1 |
| 45 | 6.42 | HA | Severe | Prophylaxis | 4 | 0 | 79.46 | 84.87 | 2 | 0 |
| 46 | 13.00 | HA | Severe | Prophylaxis | 13 | 2 | 65.35 | 78.95 | 4 | 0 |
| 47 | 10.42 | HA | Severe | On-Demand | 19 | 5 | 64.71 | 75.76 | 5 | 2 |
| 48 | 11.33 | HA | Moderate | Prophylaxis | 4 | 1 | 81.46 | 85.54 | 2 | 0 |
| 49 | 10.00 | HA | Severe | On-Demand | 16 | 8 | 60.46 | 68.29 | 4 | 2 |
| 50 | 10.83 | HA | Severe | Prophylaxis | 2 | 1 | 71.56 | 78.44 | 1 | 1 |
| 51 | 6.50 | HA | Severe | Prophylaxis | 6 | 1 | 72.53 | 79.45 | 3 | 1 |
| 52 | 10.00 | HA | Severe | Prophylaxis | 7 | 2 | 72.58 | 80.31 | 2 | 0 |
| 53 | 10.58 | HB | Severe | Prophylaxis | 3 | 1 | 76.74 | 79.15 | 1 | 0 |
| 54 | 17.92 | HA | Moderate | On-Demand | 9 | 2 | 70.49 | 78.57 | 3 | 0 |
| 55 | 4.42 | HA | Moderate | On-Demand | 8 | 1 | 72.44 | 80.13 | 2 | 1 |
| 56 | 10.42 | HA | Severe | Prophylaxis | 6 | 0 | 78.33 | 84.24 | 3 | 0 |
| 57 | 5.50 | HA | Severe | Prophylaxis | 9 | 1 | 73.72 | 83.84 | 2 | 0 |
| 58 | 7.33 | HA | Moderate | Prophylaxis | 1 | 0 | 81.58 | 84.46 | 3 | 0 |
| 59 | 4.08 | HA | Severe | Prophylaxis | 4 | 0 | 79.73 | 85.29 | 2 | 0 |

HA, hemophilia A; HB, hemophilia B.
